# Proteomic Identification of Oxidized Proteins in *Entamoeba histolytica* by Resin-Assisted Capture: Insights into the Role of Arginase in Resistance to Oxidative Stress

**DOI:** 10.1371/journal.pntd.0004340

**Published:** 2016-01-06

**Authors:** Preeti Shahi, Meirav Trebicz-Geffen, Shruti Nagaraja, Sharon Alterzon-Baumel, Rivka Hertz, Karen Methling, Michael Lalk, Serge Ankri

**Affiliations:** 1 Department of Molecular Microbiology, Bruce Rappaport Faculty of Medicine, Technion-Israel Institute of Technology, Haifa, Israel; 2 University of Greifswald, Institute of Biochemistry, Greifswald, Germany; Liverpool School of Tropical Medicine, UNITED KINGDOM

## Abstract

*Entamoeba histolytica* is an obligate protozoan parasite of humans, and amebiasis, an infectious disease which targets the intestine and/or liver, is the second most common cause of human death due to a protozoan after malaria. Although amebiasis is usually asymptomatic, *E*. *histolytica* has potent pathogenic potential. During host infection, the parasite is exposed to reactive oxygen species that are produced and released by cells of the innate immune system at the site of infection. The ability of the parasite to survive oxidative stress (OS) is essential for a successful invasion of the host. Although the effects of OS on the regulation of gene expression in *E*. *histolytica* and the characterization of some proteins whose function in the parasite's defense against OS have been previously studied, our knowledge of oxidized proteins in *E*. *histolytica* is lacking. In order to fill this knowledge gap, we performed a large-scale identification and quantification of the oxidized proteins in oxidatively stressed *E*. *histolytica* trophozoites using resin-assisted capture coupled to mass spectrometry. We detected 154 oxidized proteins (OXs) and the functions of some of these proteins were associated with antioxidant activity, maintaining the parasite's cytoskeleton, translation, catalysis, and transport. We also found that oxidation of the Gal/GalNAc impairs its function and contributes to the inhibition of *E*. *histolytica* adherence to host cells. We also provide evidence that arginase, an enzyme which converts L-arginine into L-ornithine and urea, is involved in the protection of the parasite against OS. Collectively, these results emphasize the importance of OS as a critical regulator of *E*. *histolytica*'s functions and indicate a new role for arginase in *E*. *histolytica*'s resistance to OS.

## Author Summary

Reactive oxygen species are the most studied of environmental stresses generated by the host immune defense against pathogens. Although most of the studies that have investigated the effect of oxidative stress on an organism have focused on changes which occur at the protein level, only a few studies have investigated the oxidation status of these proteins. Infection with *Entamoeba histolytica* is known as amebiasis. This condition occurs worldwide, but is most associated with crowded living conditions and poor sanitation. The parasite is exposed inside the host to oxidative stress generated by cells of the host immune system. The nature of oxidized proteins in oxidatively stressed *E*. *histolytica* has never been studied. In this report, the authors present their quantitative results of a proteome-wide analysis of oxidized proteins in the oxidatively stressed parasite. They identified crucial redox-regulated proteins that are linked to the virulence of the parasite, such as the Gal/GalNAc lectin. They also discovered that arginase, a protein involved in ornithine synthesis, is also involved in the parasite's resistance to oxidative stress.

## Introduction

Amebiasis is a parasitic infection of the intestines and is mainly caused by fecal contamination [1]. Although 90% of infected individuals are asymptomatic, amebic dysentery affects 50 million people in India, Southeast Asia, Africa, and Latin America and amebiasis is the cause of at least 100,000 deaths each year [2, 3]. Following excystation within the small intestinal lumen, trophozoites colonize the large intestine and they usually reside in the colon as a non-pathogenic commensal in most infected individuals. Due to as yet unidentified causes, these trophozoites can cause amebic dysentery, become virulent and invasive, and migrate to the liver, via the portal veins, where they cause hepatocellular damage.

Following host invasion, invading *E*. *histolytica* trophozoites are challenged by oxidative stress (OS) and nitrosative stress (NS), which originate from fluctuations in ambient oxygen tension in the intestinal lumen and the generation of reactive oxygen species (ROS) and reactive nitrogen species (RNS) by cells of the immune system. Once formed, these reactive species can oxidatively damage proteins and change their structural conformation and functional activity [4], [5], [6]. The parasite's complex response to OS involves modulation of a large number of genes which encode proteins that are associated with signaling/regulatory and repair/metabolic pathways and proteins whose exact functions are still unknown [7]. It has been recently reported that the expression of these genes is regulated by a recently identified transcription factor that binds to a specific promoter motif of hydrogen peroxide (H_2_O_2_)-responsive genes [8]. It has also been reported that those genes in *E*. *histolytica* which confer resistance to OS also contribute to its virulence [9]. Since antioxidant enzymes, such as catalase, glutathione reductase, and γ-glutamyl transpeptidase, are missing from *E*. *histolytica*'s enzyme resource [10], one of the functions of proteins, such as the 29-kDa peroxiredoxin [11] and the iron-containing peroxide dismutase [12], is to protect the parasite against OS. Since OS glycolysis is inhibited and metabolic flux is redirected towards glycerol production in oxidatively stressed *E*. *histolytica* trophozoites, these findings suggest that the glycerol synthesis pathway is a component of the parasite's metabolic antioxidative defense system [13]. Despite these informative data on the parasite's response to OS, our knowledge on the identity of oxidized proteins in *E*. *histolytica* is still incomplete. Here, we report the results of a study whose aim was to identify and to determine the biological relevance of oxidized proteins (OX) in *E*. *histolytica* using resin-assisted capture (RAC) coupled with mass spectrometry (MS) [14].

The results of this analysis revealed 154 OXs which include antioxidant proteins, cytoskeleton proteins, protein involved in translation, and transport proteins. We also found that oxidation of cysteine residues in the carbohydrate recognition domain (CRD) of the 260-kD heterodimer and multifunctional virulence factor of *E*.*histolytica*, Gal/GalNAc lectin (gl), impairs its ability to adhere to host cells. We also found that arginase, the enzyme which converts L-arginine into L-ornithine and urea, confers resistance to OS in *E*.*histolytica*.

## Methods

### Microorganisms

*E*. *histolytica* trophozoites strain HM-1:IMSS were grown under axenic conditions in Diamond's TYI S-33 medium at 37°C. Trophozoites in the exponential phase of growth were used in all experiments.

### DNA constructs

For the construction of the pJST4-arginase expression vector, arginase was amplified by polymerase chain reaction (PCR) using the primers, Arginase KpnI and Arginase BglII (table 1). The PCR product was subcloned using the pGEM-T Easy vector system (Promega) and then digested with the restriction enzymes KpnI and Bgl II. The digested DNA insert was cloned into the *E*. *histolytica* expression vector pJST4 which had been previously linearized with KpnI and Bgl II. The pJST4 expression vector (pcontrol) contains a tandem affinity purification tag for use in protein purification and identification [15]. This CHH-tag contains the calmodulin binding protein, hemagglutinin (HA), and histidine (His) residues and its expression is driven by an actin promoter. This vector was used as control in our experiment in order to exclude the possibility that the CHH tag is responsible for the phenotypes of the arginase-overexpressing strain. A previously described protocol was used to transfect *E*. *histolytica* trophozoites [16].

**Table 1:** Oligonucleotides used in this study.

Primer Name	Sequence	Direction	Restriction site-underline
Arginase Kpn1	GGTACCATGCAATTTGAAAAAGTTA	Sense	KpnI
Arginase Bgl11	AGATCTACACTTTATACCAAAAAGTG	Antisense	BglII

### Viability assay

*E*. *histolytica* trophozoites (1x10^6^) were exposed to 1 mM, 2.5 mM, 5 mM, 7 mM, or 10 mM H_2_O_2_ for 60 minutes at 37°C. At the end of the exposure, a 10-μl aliquot of each culture was stained with eosin (0.1% final concentration), and the number of living trophozoites was counted in a counting chamber under a light microscope. Resistance of the control, trophozoites overexpressing arginase and pcontrol trophozoites to OS was measured by calculating the median lethal dose (LD_50_) of hydrogen peroxide (H_2_O_2_) by linear regression analysis using Microsoft Excel. The assay was repeated three times with two replicates in each assay.

### Determination of intracellular ROS levels

Control and oxidatively stressed *E*. *histolytica* trophozoites were incubated with 0.4 mM (final concentration) 2,7-dichlorofluorescein diacetate (H2DCFDA; Sigma) for 15 minutes in the dark. The cells were washed twice in phosphate buffered saline (PBS; pH 7.4) and immediately examined under a Zeiss Axio Scope.A1 fluorescence microscope. Intracellular ROS levels were determined by measuring fluorescence intensity using the ImageJ software [17].

### Detection of OXs by OX-RAC

*E*. *histolytica* trophozoites (5x10^7^) were incubated with 2.5 mM H_2_O_2_ for 60 minutes at 37°C. At the end of the incubation, a total protein extract was prepared by lysing the oxidatively stressed trophozoites with 1% Igepal (Sigma) in PBS. OXs in the extract were detected by OX-RAC using a previously described protocol [14] with minor modifications. Briefly, the total protein extract (9 mg) was incubated in mixture of 50 mM *N*-ethylmaleimide and 2.5% sodium dodecyl sulfate (SDS) for one hour at 50°C with frequent vortexing in order to block the free thiols. The proteins were then precipitated with three volumes of cold 100% acetone and incubated at -20°C for 20 minutes. The mixture was centrifuged at 1820g for five minutes, and the pellet was then washed three times with 70% acetone (3 volumes) and then resuspended in HENS buffer which contains 100 mM HEPES, 1 mM EDTA, 0.1 mM neocuproine, and 1% SDS). The resuspended samples were added to 80 μl thiopropyl sepharose 6B resin (GE Healthcare) in the presence or absence of dithiothreitol (DTT, final concentration 10 mM). DTT is a reducing agent, which enables the oxidized thiol group of cysteine to bind to the resin by forming disulfide bonds between the reduced thiol groups of the proteins and the thiol group of the resin. The samples were rotated in the dark at room temperature for 1–2 hours, and then overnight at 4°C. The resin was washed four times with 1 ml HENS buffer, and then twice with 1 ml HENS/10 buffer (1:10 HENS buffer). Captured proteins were eluted with 30 μl HENS/10 buffer which contained 100 mM 2-mercaptoethanol for 20 minutes at room temperature, and the proteins in each eluent were resolved on a 12.5% SDS-PAGE gel. Each gel was then stained with silver (Pierce Silver Stain) and each gel slice was independently analyzed by MS.

### In-gel proteolysis for MS-based protein identification

The proteins in each gel slice were reduced with 2.8 mM DTT (60°C for 30 minutes), modified with 8.8 mM iodoacetamide in 100mM ammonium bicarbonate in the dark at room temperature for 30 minutes, and digested overnight in 10% acetonitrile and 10 mM ammonium bicarbonate with modified trypsin (Promega-Biological Industries, Israel) at 37°C. The resulting peptides were resolved by reverse-phase chromatography on 0.075 x 200-mm fused silica capillaries (J&W Scientific, Agilent Technologies, Israel) packed with Reprosil reversed phase material (Dr. Maisch GmbH, Germany). The peptides were eluted at flow rates of 0.25 μl/min on linear gradients of 7–40% acetonitrile in 0.1% formic acid for 95 minutes followed by eight minutes at 95% acetonitrile in 0.1% formic acid. MS was done by an ion-trap mass spectrometer (Orbitrap, Thermo) in a positive mode using a repetitively full MS scan followed by collision-induced dissociation (CID) of the seven most dominant ions selected from the first MS scan. The MS data was analyzed using the Proteome Discoverer software version 1.3 which searches the Ameba section of the NCBI-NR database and the decoy databases (in order to determine the false discovery rate (FDR)) using the Sequest and the Mascot search engines.

### Classification of OXs according to their protein class

The OXs were classified according to their protein class using the PANTHER software (Protein ANalysis THrough Evolutionary Relationships) Classification System (http://www.pantherdb.org/) [18].

### Adhesion assay

The adhesion of oxidatively stressed trophozoites to HeLa cell monolayers was measured using a previously described protocol [19]. Briefly, trophozoites (2×10^5^) were exposed to 2.5 mM H_2_O_2_ for 20 minutes at 37°C, washed twice with Dulbecco's modified Eagle's medium (DMEM) without serum, added to wells that contained fixed HeLa monolayers in 1 ml of DMEM without serum, and incubated for 30 minutes at 37°C. The number of adherent trophozoites was determined by counting the number of trophozoites that remained attached to the HeLa cells after gentle decanting (twice) of the non-adherent trophozoites with warm (37°C) DMEM under a light microscope.

### Determination of *E*. *histolytica* motility

The Costar Transwell System (8-μm pore size polycarbonate membrane, 6.5-mm diameter, Corning Inc, Corning, NY, USA) was used to determine trophozoite motility [20]. Briefly, 24-well culture plate was filled with serum-free Diamond’s TYI-S-33 medium (500-μl per well). A transwell insert was then inserted into each well. Control and oxidatively stressed (2.5 mM and 1 mM for one hour at 37°C) trophozoites were washed three times in serum-free Diamond’s TYI-S-33 medium, and then suspended in serum-free Diamond’s TYI-S-33 medium. A 500-μl aliquot of the suspension (26x10^5^ trophozoites/ml) was then loaded into the transwell inserts. The 24-well culture plate containing the transwell inserts was then placed in anaerobic bags (Mitsubishi Gas Chemical Company, Inc., Tokyo, Japan), and incubated for three hours at 37°C. At the end of the incubation, the inserts and culture medium were removed from the 24-well culture plate, and trophozoite migration was determined by counting the number of trophozoites that were attached to the bottom of the wells of the 24-well culture plate.

### Purification of Gal/GalNAc lectin by affinity chromatography

Gal/GalNAc lectin was purified using a previously described protocol [21]

### Oxidation of purified Gal/GalNAc lectin

Aliquots (5 μg) of purified Gal/GalNAc lectin were incubated with either 0.1 mM or 2.5 mM H_2_O_2_ for ten minutes at 37°C. The Gal/GalNAc lectin was then incubated with 10 μl D-galactose-coated agarose beads (Thermo Scientific-Pierce) overnight at 4°C. At the end of the incubation, the beads were washed in 20 volumes of PBS and then boiled in Laemmli sample buffer. The amount of Gal/GalNAc lectin that was released from the beads was determined using SDS-PAGE gel electrophoresis and silver staining (Pierce).

### Detection of oxidized Gal/GalNac lectin

Aliquots (5 μg) of purified Gal/GalNac lectin [21] were treated with 1 mM H_2_O_2_ for ten minutes at room temperature in order to introduce carbonyl groups into protein side chains. Using the OxyBlot Protein Oxidation Detection Kit (Millipore, Israel) [22], the carbonyl groups are derivatized with 2,4-dinitrophenylhydrazine (DNPH). The DNPH-treated Gal/GalNac lectin was separated by SDS-PAGE, transferred onto a nitrocellulose membrane and then detected by a specific antibody against the dinitrophenyl (DNP) moiety of the OXs. The nitrocellulose membrane has been stripped and probed with a polyclonal Gal/GalNAc lectin antibody (a kind gift from of N. Guillen, Pasteur Institute, Paris, France) to confirm that equal amounts of purified Gal/GalNac lectin were loaded on the gel.

### Determination of protein synthesis by surface sensing of translation (SUnSET)

SUnSET was performed using a previously described protocol [23]. Briefly, trophozoites (2x10^6^/ml) that were treated with 2.5 mM H_2_O_2_ for 15 minutes at 37°C and untreated control trophozoites were incubated with 10 μg/ml puromycin (Sigma), a structural analog of tyrosyltRNA, for 20 minutes. For pretreatment of the trophozoites with cycloheximide (Sigma), the trophozoites were incubated with 100 μg/ml cycloheximide for five minutes before adding puromycin. The trophozoites were lysed using 1% Igepal (Sigma) in PBS. Puromycin was detected by immunoblotting using a monoclonal puromycin antibody (12D10 clone, Millipore). Protein quantification was measured by band intensity (densitometry) using ImageJ software [17].

### Determination of arginase activity

Arginase activity in *E*. *histolytica* crude lysate was spectrophotometrically measured by quantifying the amount of urea that is generated when L-arginine is hydrolyzed by arginase using a previously described protocol [24]. Briefly, 10^4^ trophozoites were dissolved in 100 μl of 0.1% Triton X-100 (Sigma) in the presence of 50 μM L-3-carboxy-2,3-trans-epoxypropionyl-leucylamido(4-guanidino)-butane (E-64) (Sigma), a cysteine protease inhibitor. The lysate (50 μl) was mixed with 50 μl of Tris-HCl (50 μM; pH 7.5) which contained 10 mM MnCl_2_, and then activated by heating for ten minutes at 55°C. The hydrolysis of L-arginine by arginase was initiated by adding 25 μl L-arginine (0.5 M; pH 9.7) to a 25 μl aliquot of activated lysate. After a 30-minutes incubation at 37°C, the reaction was stopped by adding 400 μl of an acid solution mixture (H_2_SO_4_: H_3_PO_4_: H_2_O = 1: 3: 7). The urea concentration in the mixture was measured at 570 nm after adding α-isonitrosopropiophenone (25 μl, 9% in absolute ethanol) to the mixture, heating the mixture for 45 minutes at 100°C, and incubating the mixture in the dark for ten minutes at room temperature.

### HPLC analysis of amino acids in culture supernatants and intracellular amino acids

The amino acid levels in culture supernatants were analyzed by high-performance liquid chromatography (HPLC) using a previously described protocol [24] in which the proteins in the culture supernatant are first precipitated with methanol, and the amino acids are derivertized using o-phthalaldehyde (OPA) in an alkaline medium. Briefly, 200 μl of culture supernatants are added to an 800-μl mixture of methanol and internal standard (homocysteic acid). After centrifugation, the samples are loaded into the HPLC autosampler, which converts the samples to fluorescent derivatives (by mixing them with OPA) before their injection into the columns (C-18). A JASCO FP 1520 fluorescence detector at an excitation wavelength of 360 nm with emission detection at 455 nm was used to separate, detect, and quantify the fluorescent derivatives. For quantification of the intracellular amino acids, trophozoites (10^7^) were lysed in 1 ml of trichloroacetic acid (TCA) 10% for 30 minutes at 4°C, and centrifuged, and the pH of the supernatants was adjusted to 12 using 10N NaOH. The amino acid concentration in the supernatants was then measured by HPLC on two biological replicates.

### ^1^H-NMR spectroscopic analysis of putrescine in trophozoite lysates

The quantification of putrescine in the trophozoite lysates was performed using ^1^H- nuclear magnetic resonance (NMR) spectroscopy as previously described [25, 26]. Two biological replicates were used for each measure. Briefly, 400 μl H_2_O was used to dissolve the lysate and mixed with 200 μl buffer solution containing the internal standard TSP (3-trimethylsilyl-[2,2,3,3-D_4_]-1-propionic acid) (Sigma-Aldrich). All NMR spectra were obtained at 600.27 MHz at a temperature of 310 K, using a Bruker Avance-II 600 NMR spectrometer operated by TOPSPIN 3.2 software (Bruker Biospin GmbH). Spectral referencing was done relative to the TSP signal (final concentration 0.33mM). Data analysis (identification) was done using AMIX v3.9.14 software (Bruker Biospin GmbH) as previously described [25, 26]. The quantification was performed using the software package CHENOMX (version 8.1).

## Results

### Characterization of oxidized proteins in oxidatively stressed *E*. *histolytica* trophozoites

**Fig 1:**
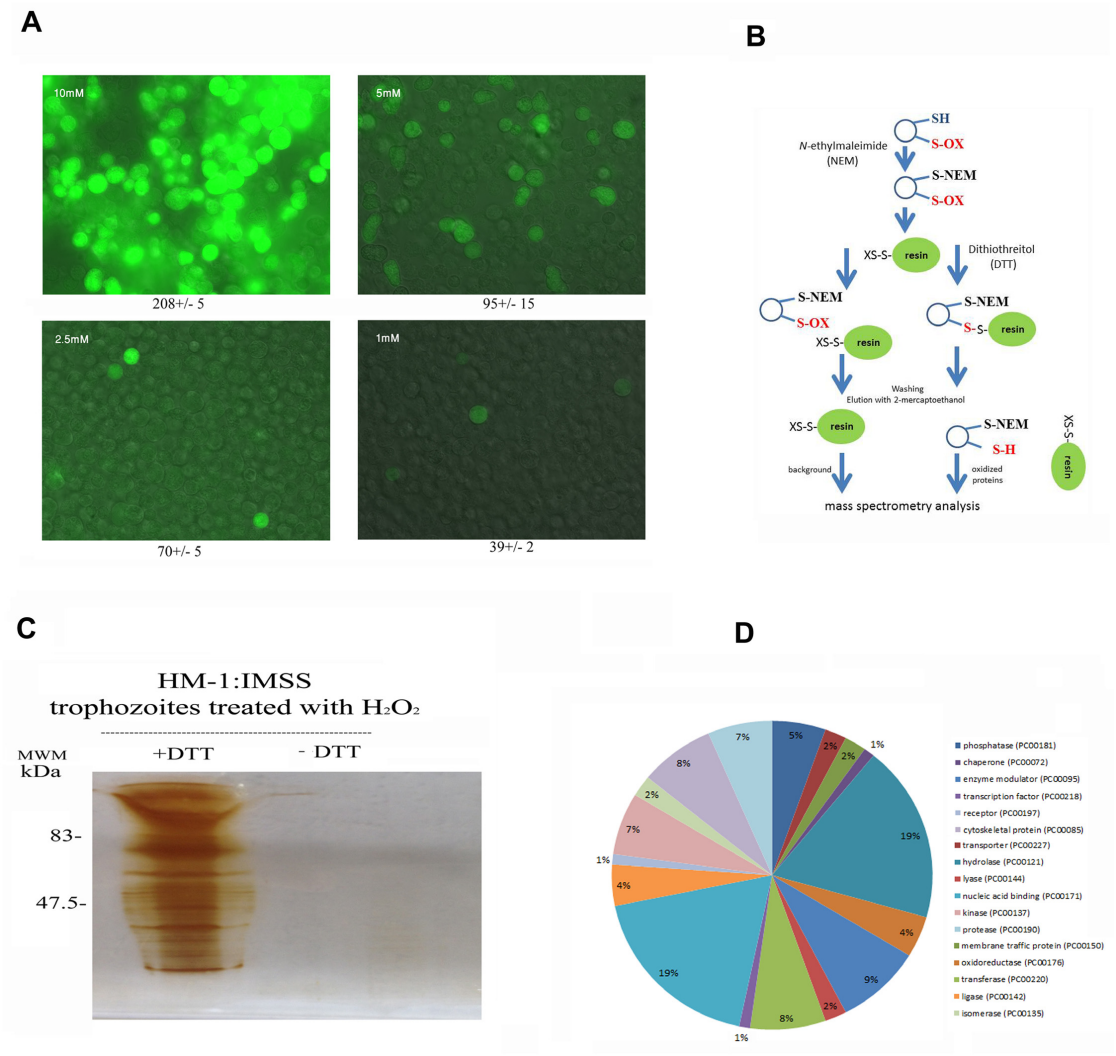
Analysis of oxidized proteins in *E*. *histolytica* after resin-assisted capture. A. Detection of intracellular ROS level. Control and oxidatively stressed trophozoites were incubated with 0.4 mM (final concentration) 2,7-dichlorofluorescein diacetate for 15 minutes in the dark and then examined by fluorescence microscopy. The number in the top left side of each picture indicates the concentration of H_2_O_2_. The number in the bottom of each picture indicates the mean fluorescence for 20 cells according to a measure performed by image J. B. Methodology of OX-RAC. The scheme has been adapted from [14]. C. Silver staining of oxidized proteins. *E*. *histolytica* trophozoites strain HM-1:IMSS were treated with H_2_O_2_ for 60 minutes at 37°C and a total protein lysate was prepared by lysing the H_2_O_2_-treated trophozoites with 1% Igepal (Sigma) in phosphate buffered saline. The oxidized proteins in the cell lysates were subjected to resin-assisted capture (RAC) in the presence of 10 mM DTT (+DTT) or the absence of DTT (–DTT). D. Functional categories of all oxidized proteins. Oxidized proteins in *E*. *histolytica* were classified according to the protein class they encode using PANTHER sequence classification tool.

**Fig 2:**
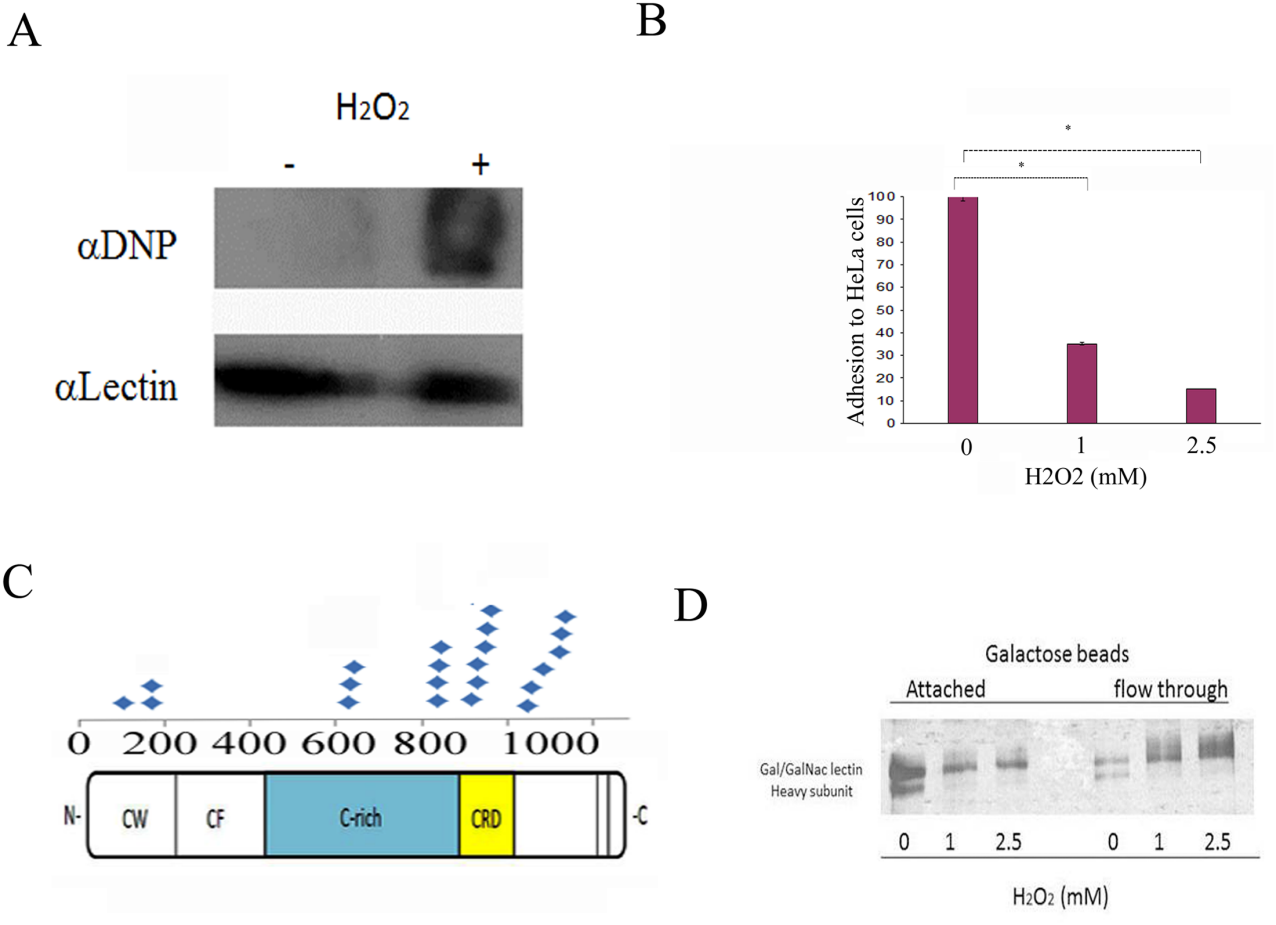
H_2_O_2_ inhibits the adhesion of *E*. *histolytica* to HeLa cells. A. Confirmation of OX-RAC data about the oxidation of the Hgl by using the OxyBlot protein oxidation detection kit. This figure displays a representative result from two independent experiments. B. *E*. *histolytica* trophozoites strain HM-1:IMSS were grown in Diamond’s TYI-S-33 medium and exposed to H_2_O_2_ for 20 minutes before their incubation with a fixed HeLa cell monolayer for 30 minutes at 37°C. Data are expressed as the mean ± standard deviation of three independent experiments that were performed in triplicate. The adhesion of the H_2_O_2_-treated trophozoites was significantly different (p<0.05) from the control (100%) according to the results of an unpaired Student’s t-test in which statistical significance was set at 5%. C. Repartition of the carbamidomethylated cysteine residues in the Hgl as an indication of their oxidation status. These residues were mostly located in the cysteine-rich region (C-rich) in the carbohydrate recognition domain (CRD) and in the cysteine-tryptophan domain (CW) and were absent in the cysteine-free domain (CF). D. Dose-dependent inhibition of Gal/GalNAc lectin binding to D-galactose-coated agarose beads by H_2_O_2_. Gal/GalNAc lectin was purified by D-galactose affinity chromatography and incubated with different concentrations of H_2_O_2_ for 10 minutes at 37°C. The binding to galactose-coated agarose beads was determined by SDS-PAGE gel electrophoresis and silver staining. This figure displays a representative result from two independent experiments.

When *E*. *histolytica* trophozoites strain HM-1:IMSS were incubated with 1 mM, 2.5 mM, 5 mM, 7 mM, or 10 mM H_2_O_2_ for 60 minutes at 37°C, the calculated LD50 of H_2_O_2_ is 5.5 ± 0.1 mM (Table 2) and the intracellular ROS levels in living trophozoites are high (Fig 1A). Based on these results, we selected 2.5 mM as the H_2_O_2_ concentration to oxidatively stress trophozoites in our various assays because this concentration is not lethal (85% of the trophozoites are viable; S1 Fig), the intracellular ROS levels are relatively low (Fig 1A), and OXs are formed (this work). We then used OX-RAC coupled to label-free quantification LC-MS for the detection and quantification of OXs in the lysate of oxidatively stressed trophozoites (Fig 1B). A protein was considered to be oxidized when its relative amount in the DTT-treated lysates was at least two times greater than that in the untreated lysates (Fig 1C). We identified 154 proteins that met this condition (S1–S3 Tables). These 154 proteins were then classified (Fig 1D) using PANTHER sequence classification tool [27, 28]. The protein classes were phosphatases (exemplified by phosphoinositide phosphatase (EHI_141860); transporters (exemplified by plasma membrane calcium-transporting ATPase, EHI_030830); membrane traffic proteins (exemplified by putative vacuolar sorting protein, EHI_025270); chaperones (exemplified by Hsc70-interacting protein, EHI_158050); hydrolases (exemplified by arginase, EHI_152330); oxidoreductases (exemplified by superoxide dismutase, EHI_159160); enzyme modulators (exemplified by Ras family GTPase, EHI_058090); lyases (exemplified by tRNA pseudouridine synthase, EHI_151650); transferases (exemplified by histone acetyltransferase, EHI_152010); nucleic acid binding proteins (exemplified by 13 kDa ribonucleoprotein-associated protein, EHI_104600); ligases (exemplified by ubiquitin-conjugating enzyme family protein, EHI_070750); kinases (exemplified by galactokinase, putative, EHI_094100); isomerases (exemplified by cysteine synthase A, EHI_024230); cytoskeletal proteins (exemplified by actin-binding protein, cofilin/tropomyosin family, EHI_168340) and proteases (exemplified by methionine aminopeptidase, EHI_126880). In order to evaluate the consistency of MS-based identification of OXs, the purified heavy subunit of Gal/GalNac lectin (Hgl) was exposed to 1 mM H_2_O_2_ for ten minutes and its oxidation was confirmed independently by using the OxyBlot kit. The presence of carbonyl groups was detected using a specific antibody which recognizes the DNP moiety in the purified lectin that has been exposed to H_2_O_2_ and treated with DNPH (Fig 2A). As expected, carbonyl groups were not detected in purified lectin that was not exposed to H_2_O_2_ and treated with DNPH.

**Table 2:** LD_50_ of H_2_O_2_ in *E*. *histolytica* trophozoites strain HM-1 IMSS, trophozoites overexpressing arginase and pcontrol trophozoites.

Strain	LD_50_ of H2O2 (mM)
*E*. *histolytica* trophozoites strain HM-1:IMSS	5.5±0.1
pcontrol trophozoites	5.1±0.1
arginase-overexpressing *E*. *histolytica* trophozoites	6.2±0.08

Data are expressed as the mean and standard error of mean of three independent experiments that were performed in duplicate. The means of the different groups for three independent experiments were compared using an unpaired Student’s t test. The LD_50_ of HM-1 IMSS or pcontrol trophozoites was significantly different (p<0.05) from that of the arginase-overexpressing trophozoites.

According to the PANTHER statistical overrepresentation test which compares classifications of multiple clusters of lists to a reference list, very significant enrichment (fold enrichment > 5 and P > 2.42x10^-6^) was found for proteins involved in the process of translation, such as the 60S ribosomal protein L9-like protein (EHI_193080) and the 13 kDa ribonucleoprotein-associated protein (EHI_104600).

The results of a previous study showed that (a) gene expression of oxidatively stressed *E*. *histolytica* trophozoites triggers a stress response in which 185 genes are upregulated and 102 genes are downregulated and (b) these genes are involved in signaling/regulatory processes, metabolic/repair processes, energy metabolism, the stress response, and transport [7]. We found that some of the OXs in the oxidatively stressed trophozoites are involved in the stress response, transport, and metabolism. Of all the proteins that were found to be oxidized and all the genes whose expression had changed in the oxidatively stressed trophozoites, EHI_179080 (sulfate adenylyltransferase) was the only common gene.

Cysteine residues are the predominant targets of oxidation or S-nitrosylation in redox-sensitive proteins. We have recently identified 142 S-nitrosylated (SNO)-proteins in *E*. *histolytica* after its exposure to NO [21]. 21 proteins were shared in our OX-RAC and SNO-RAC analysis (Table 3). The shared proteins include rubrerythrin, protein disulfide isomerase and iron-containing superoxide dismutase which have been associated with resistance to OS [12, 27–29] and the Gal/GalNac lectin, a cell surface protein which is involved in binding of *E*. *histolytica* troohozoites to host cells [17,18].

**Table 3:** Common proteins in the OX-RAC analysis (this study) and SNO-RAC analysis [21].

Accession	Description
183232225	Plasma membrane calcium-transporting ATPase
67469645	Hsc70-interacting protein
67481145	Rab family GTPase
67474574	hypothetical protein
67462443	NA modification enzymes, MiaB-family
67472683	rubrerythrin
67469707	Rho family GTPase
67477041	Ras family GTPase
67465045	60S ribosomal protein L18a
5689218	serine acetyltransferase
67482289	peptidyl-prolyl cis-trans isomerase
67465747	60S ribosomal protein L7
67481543	40S ribosomal protein S13
183234048	alcohol dehydrogenase
67465285	Iron-containing superoxide dismutase
67464751	ARP2/3 complex 20 kDa subunit
67465767	Galactokinase
67481663	Hgl
183232524	adenylyl cyclase-associated protein
67478039	hypothetical protein
52421800	protein disulfide isomerase

### Regulation of the Gal/GalNAc Lectin by OS

In order to gain information on the consequence of oxidation on the activity of some of the proteins that were identified in the OX-RAC analysis, we decided to focus our analysis on the function of the oxidized Gal/GalNac lectin. The Gal/GalNac lectin consists of Hgl (170 kDa) and a light subunit (Lgl) (35/ 31 kDa). Hgl mediates *E*. *histolytica* adherence, and indirect evidence suggests that Lgl plays a role in *E*. *histolytica* virulence [13,19]. The occurrence of Hgl among the OXs (see S2 Table) and SNO proteins ([21] and Table 3) suggests that OS and NS affect the parasite's adherence. In order to test this hypothesis, we compared the ability of untreated and oxidatively stressed trophozoites to adhere to HeLa cells [13]. We found that the binding of the oxidatively stressed trophozoites to the HeLa cell monolayer was significantly less than that of the untreated trophozoites (65% and 85%; Fig 2B). Previous research results show that the cysteine-rich region (CRR) of the Hgl and the CRD are important for the binding activity of the lectin [19, 20]. Our MS analysis of OXs indicates that many carbamidomethylated cysteine residues, that possibly represent oxidized cysteines, are located in the CRR and in the CRD of Hgl (Fig 2C). When we investigated the binding ability of oxidized Gal/GalNAc lectin, we observed that H_2_O_2_ prevents the binding of the Gal/GalNAc lectin to the galactose beads (Fig 2D).

### Effect of OS on *E*. *histolytica* motility

**Fig 3:**
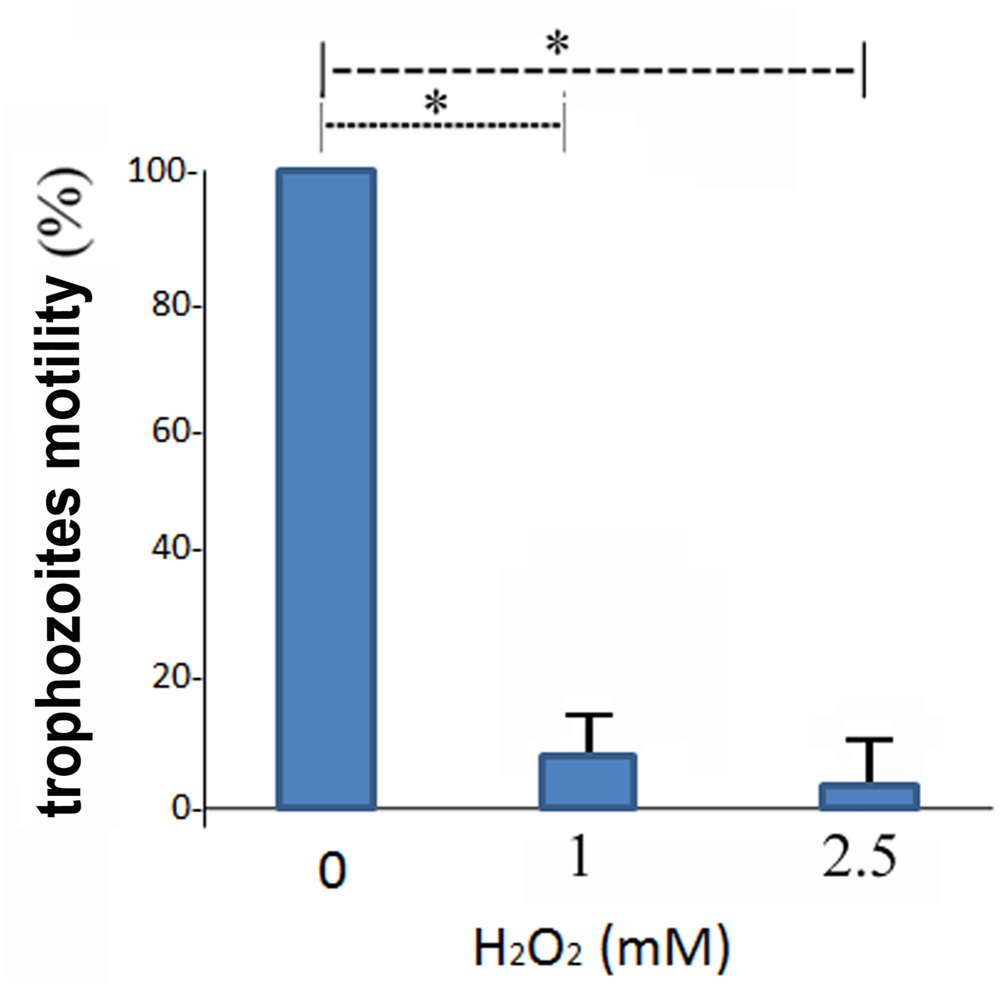
Effect of H_2_O_2_ on *E*. *histolytica* motility. The motility of control and oxidatively stressed *E*. *histolytica* trophozoites was investigated using a transwell assay (see material and methods for details). The motility of control trophozoites that were not exposed to H_2_O_2_ was set at 100%. Data are expressed as the mean ± standard deviation of three independent experiments that were repeated twice. The motility of the H_2_O_2_-treated trophozoites was significantly different (p<0.05) from that of the controls according to the results of an unpaired Student’s t-test in which statistical significance was set at 5%.

Motility is important for *E*. *histolytica* survival and pathogenicity, and requires a dynamic actin cytoskeleton [30]. The presence of cytoskeleton-associated proteins, such as the ARP2/complex 20kDa subunit, among the OXs (S2 Table) and the SNO- proteins ([21] and Table 3) suggests that the parasite's motility is redox-regulated. We tested this hypothesis by comparing the migration of control and oxidatively stressed trophozoites: the migration of the oxidatively stressed trophozoites was significantly less than that of the control trophozoites (Fig 3).

### Effect of OS on protein synthesis

**Fig 4:**
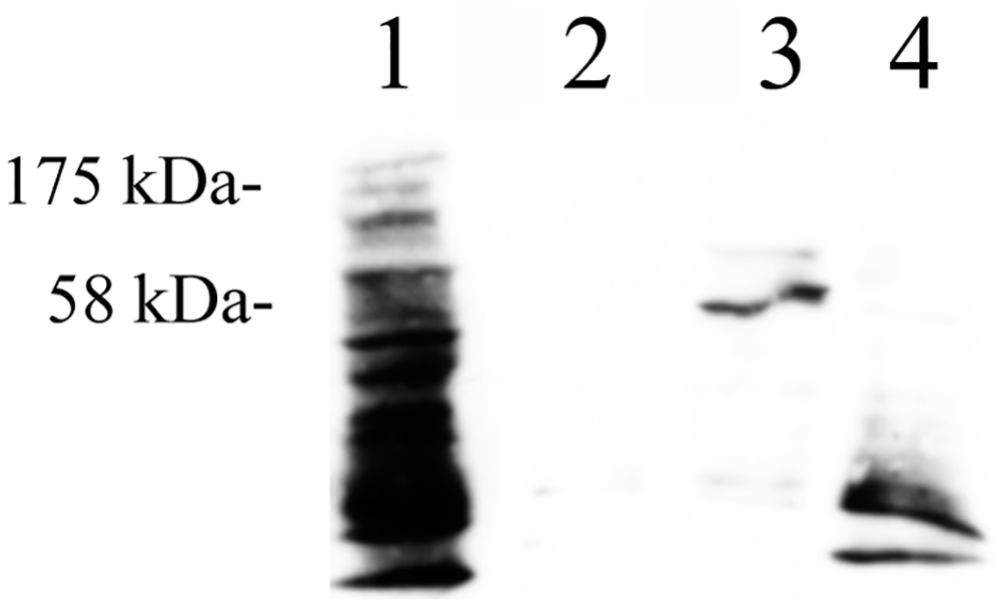
Effect of H_2_O_2_ on *E*. *histolytica* protein synthesis measured using puromycin-labeled proteins. Lane 1: Trophozoites were treated with 10 μg/ml puromycin (lane 1), not treated with puromycin (lane 2), treated with 2.5 mM H_2_O_2_ for 15 minutes and then labeled with 10 μg/ml puromycin for 20 minutes (lane 3) and treated with cycloheximide (100 μg/ml) before puromycin labeling (lane 4). The extracts were separated by denaturing electrophoresis and analyzed by immunoblotting with a monoclonal puromycin antibody 12D10 clone. An actin immunoblot is shown as the loading control.

Inhibition of translation is a typical response of cells exposed to stress conditions [31, 32], and such inhibition may avoid constant gene expression during error-prone environments. We recently reported that NS inhibits protein synthesis in *E*. *histolytica* [33]. The presence of the 60S ribosomal protein L7, the 60S ribosomal protein L18a and the 40S ribosomal protein S13 among the OXs (S2 Table) and SNO proteins ([21] and Table 3) suggest that oxidation is similar to S-nitrosylation [21], in that it regulates the translation of proteins in the parasite. In order to test this hypothesis, we used SUnSET [23][33, 34] to determine protein synthesis. We found that protein synthesis is strongly inhibited in oxidatively stressed trophozoites and that the level of inhibition is comparable to that found in cyclohexamide-treated trophozoites (Fig 4) [35].

### Overexpression of arginase confers resistance to OS

**Fig 5:**
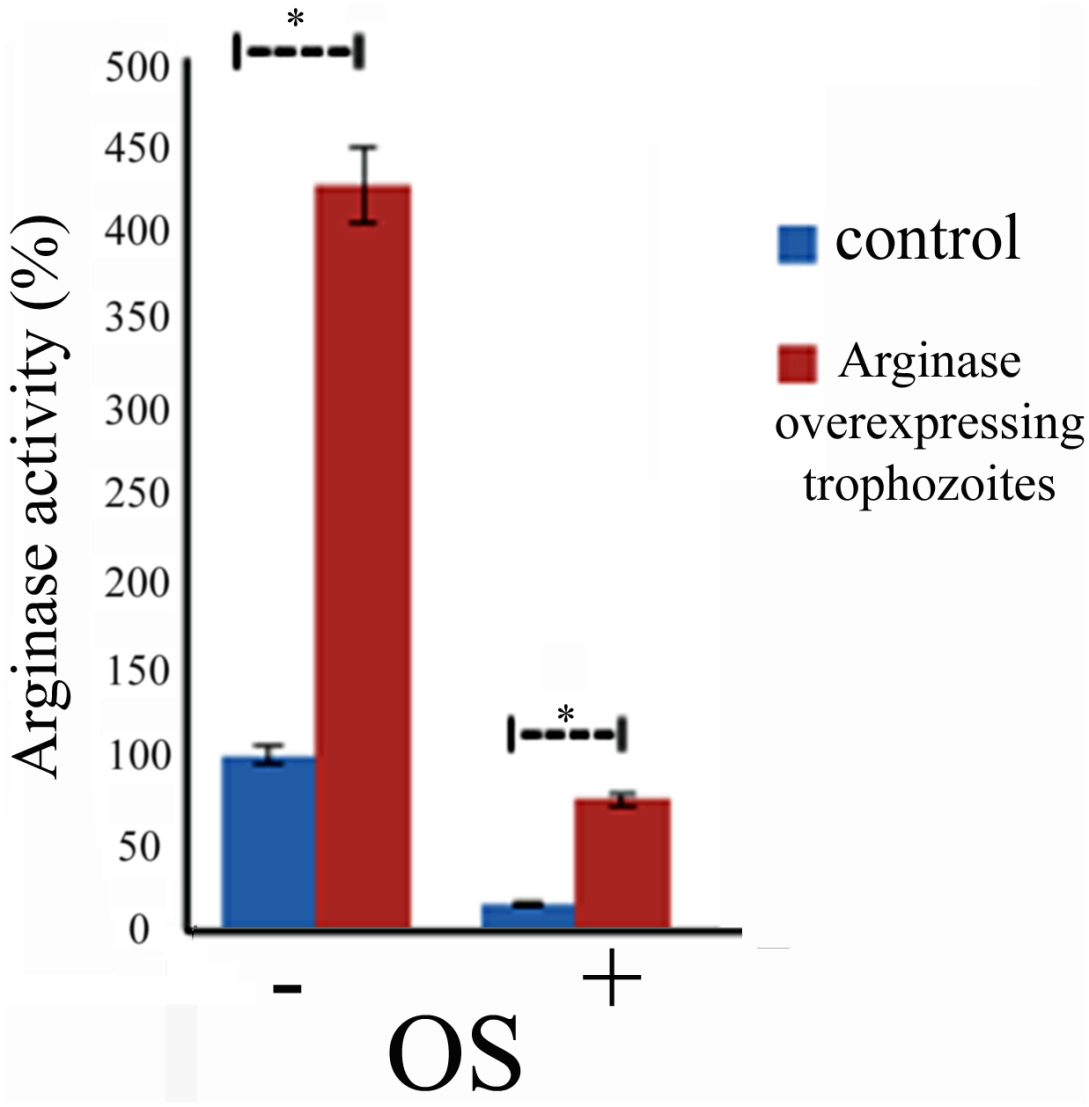
Overexpression of arginase confers resistance to OS. Arginase activity was measured in crude lysates that were prepared from control and arginase-overexpressing *E*. *histolytica* trophozoites exposed or not to OS. Data are expressed as the mean ± standard deviation of three independent experiments that were repeated twice. The arginase activity of the arginase-overexpressing trophozoites was significantly different (p<0.05) from the control (100%) according to the results of an unpaired Student’s t-test. The arginase activity of the oxidatively stressed arginase-overexpressing trophozoites was significantly different (p<0.05) from the oxidatively stressed control according to the results of an unpaired Student’s t-test. Arginase activity in the control trophozoites was 120 μmol urea/min/mg proteins.

We previously reported that the enzymatic conversion of L-arginine to L-ornithine is an significant source of L-ornithine for *E*. *histolytica* and that arginase activity is essential for the resistance of the parasite to NS [24]. The presence of L-arginase among the OXs raises questions about the regulation of its activity by OS and its involvement in the resistance of the parasite to OS. In order to obtain information about the effect of OS on arginase activity, we measured arginase activity in control and oxidatively stressed trophozoites using a previously described assay [24]. We found that arginase activity is markedly inhibited (90% inhibition) in the oxidatively stressed trophozoites (Fig 5)_._ In order to establish whether arginase gene expression is involved in the resistance of the parasite to OS, we decided to upregulate its expression (Fig 5). We detected modest arginase activity in arginase-overexpressing trophozoites and no arginase activity in control trophozoites that were exposed to 2.5 mM H_2_O_2_ for 15 minutes (Fig 5). We found that the arginase-overexpressing trophozoites are more resistant to OS (LD_50_ 6.2±0.08 mM) than the control trophozoites (IC_50_ LD_50_ 5.1±0.1 mM) (table 2). We also found that the intracellular arginine concentration in the arginase-overexpressing trophozoites (53 ± 5 μM) was significantly lower than that in the control trophozoites (136±9 μM). No significant difference in the intracellular ornithine concentration in the arginase-overexpressing trophozoites (616±7 μM) and the control trophozoites (577±15 μM) was detected. In contrast, we found that the intracellular concentration of putrescine in the arginase-overexpressing trophozoites (293±20 nmol/mg protein) was significantly higher than that of the control trophozoites (123±3 nmol/mg protein).

## Discussion

The current understanding of the antiamebic effect of OS [36] has greatly beneficiated from previous omics studies [13] [7] on the response of *E*. *histolytica* to OS. However, these studies did not address the nature of OXs in oxidatively stressed *E*. *histolytica* trophozoites. We decided to use OX-RAC coupled to MS [14] to generate new data about OXs in *E*. *histolytica*. Some of the proteins that we identified in our OX-RAC analysis of OXs are of particular interest because (i) they have an important function in the parasite's physiology and/or virulence and (ii) their activity is regulated by OS in other organisms.

The first notable OX that we identified in our OX-RAC analysis is the cysteine-rich (C-rich) region (amino acids 356–1143) and a CRD (amino acids 895–998) of Hgl [20]. We recently showed that S-nitrosylation of cysteine residues in the CRD inhibits the galactose binding activity of the Gal/GalNAc lectin and contributes to the reduced binding of NO-treated trophozoites to their target cells [21]. In this study, we found that carbamidomethylated cysteine residues are also located in the CRR and the CRD of the lectin. This result suggests that oxidation of these cysteines is responsible for the loss of galactose-binding activity of the Gal/GalNAc lectin.

We found that cytoskeletal proteins are oxidized in oxidatively stressed trophozoites. For its invasion into the host's tissues, *E*. *histolytica* relies on its dynamic actin cytoskeleton [37], [38]. Oxidation of the actin cytoskeleton can modulate its cellular functions in mammalian cells [39] [40] [41] and the results of proteomics studies in human peripheral blood mononuclear cells have previously identified actin as an oxidation target [42]. Actin oxidation inhibits its polymerization [43] [44] and leads to cytoskeletal rearrangements [45]. According to previous reports, the cysteines in actin are some of the most susceptible targets of oxidation [45] and their oxidative modification is the likely cause of cytoskeletal rearrangements in oxidatively stressed mammalian cells [46] [47] [48]. Actin is a highly conserved protein among species [49]. The actin of rats and *E*. *histolytica* share a number of oxidized amino acid residues (Met_44_, Met_47_) which are located in actin−actin contact regions [50] suggesting that actin oxidation is part of the process that results in the inhibition of the transwell migration that we observed in the oxidatively stressed trophozoites.

We detected strong enrichment of OXs which are components of the parasite's translational machinery, such as ribosomal proteins and elongation factors. This finding is in agreement with the results of a recent study in yeast cells, in which it was reported increases in ribosomal proteins and elongation factors due to oxidative thiol modifications following a short-term exposure to H_2_O_2_ [51]. At first glance, this finding is also in agreement with the inhibition of protein synthesis in oxidatively stressed *E*. *histolytica* trophozoites (this work) and mammalian cells [52]. However, a recent report suggests that the oxidation of components of the translational machinery is not the direct cause of the inhibition of protein synthesis, but rather a global, enzymatic downregulation of almost all tRNA species in OS [53]. It will be interesting to determine whether the same enzymatic downregulation of tRNA species also occurs in oxidatively stressed *E*.*histolytica*.

An additional group of OXs which we identified are the oxidoreductases, which includes the iron-containing superoxide dismutase. OS induces the expression of this protein [12] and the protein can form adducts with metronidazole metabolites [54]. In *E*.*coli*, iron-containing superoxide dismutase is inactivated by H_2_O_2_ via a reaction of H_2_O_2_ with the iron at the active site that generates a potent oxidant which attacks tryptophan residues [55]. It has been recently showed for *Trypanosoma* superoxide dismutase (Fe-SODB) that Cys(83) in Fe-SODB acts as an electron donor that repairs the tyrosyl radical (Tyr35-O•) via intramolecular electron transfer in order to prevent inactivation of Fe-SODB by peroxynitrite, which is produced by immunostimulated macrophages [56]. Interestingly, this Cys(83) is conserved in *E*. *histolytica*'s iron-containing superoxide dismutase and Cys(83) was detected by MS as a carbamidomethylated cysteine residue which strongly suggest that it has been oxidized. Based on this data, it is tempting to speculate that Cys(83) in *E*. *histolytica*'s iron-containing superoxide dismutase is crucial for protecting the protein against inactivation by H_2_O_2._

The phosphatases are another group of OXs which we identified in our OX- RAC analysis. This group includes the protein-tyrosine phosphatases (PTP). Two PTPs have been cloned from *E*.*histolytica*, EhPTPA and EhPTPB. EhPTPA but not EhPTB is strongly up-regulated in trophozoites that have been recovered from amebic liver abscesses suggesting that EhPTPA is involved in the parasite’s virulence. [57].

The regulation of phosphatase function by OS is well documented [58–60]. PTPs are characterized by an 11-residue signature motif (I/V)HCXAGXXR(S/T/G) in their active site [61]. The oxidation of the catalytic cysteine in this signature sequence leads to their reversible inactivation. Remarkably, the cysteine at the catalytic site of *E*. *histolytica*'s EhPTPA (IKGIKLNGPPIIHCSAGLGRSGTFI) was detected as a carbamidomethylated cysteine residue by MS, and this finding strongly suggests that it has been oxidized. Accordingly, we surmise that *E*. *histolytica* EhPTPA activity will be inhibited by OS and in the future, it will be interesting to study the consequence of this inhibition on the parasite’s virulence.

Another group of OXs which we identified in our OX- RAC analysis are the transport proteins and this group includes plasma membrane calcium-transporting ATPase. Ca^2+^-ATPases regulate intracellular calcium levels in eukaryotic cells and are thus essential to the correct functioning of the cell machinery. Calcium is also important in numerous cellular processes in *E*.*histolytica*, such as development and virulence [62, 63]. Five putative Ca^2+^-ATPases, which could be important in the regulation of the cytoplasmic calcium concentration, have been recently identified in *E*. *histolytica* [64]. Ca^2+^-ATPases are very sensitive to OS and undergo functional and conformational changes when exposed to oxidants [65]. It is possible that the same observation applies to the *E*. *histolytica* Ca2+-ATPases identified in our Ox-RAC analysis of oxidized proteins.

Arginase (EHI_152330) is a hydrolase which we identified as being oxidized in our OX-RAC analysis. Whereas arginase activity has been associated with resistance to NS in various unicellular parasites including *E*. *histolytica* [24, 66, 67], its involvement in OS resistance has never been investigated. In this study, we found that overexpression of arginase protects *E*. *histolytica* against OS. The inhibitory effect of OS on arginase activity has also been found in *Helicobacter pillory* [68]. Only three cysteine groups are present in Eharginase and their role in the enzyme's activity is unknown. A clue about their role may be deduced from the results of a previous study which found that arginase activity was inhibited in erythroleukemic K562 cells that were exposed to aurothiomalate, a gold analog that can specifically react with a protein sulphydryl group to form a thiol-gold adduct [69]. Since this finding suggests that one or more of the cysteine residues in arginase are essential for its activity, we surmise that Eharginase activity is also dependent on the presence of cysteine residues.

A possible mechanism to explain why arginase overexpression protects *E*. *histolytica* against OS involves the strong reduction of intracellular arginine that we detected in the arginase overexpressing trophozoite. We presume that this reduction might be due to the conversion of arginine into ornithine by the excess of arginase and from ornithine into putrescine by ornithine decarboxylase (ODC) [70–72]. This presumption is supported by the higher intracellular concentration of putrescine found in the arginase-overexpressing trophozoites compared with that of the control trophozoites. ODC is the only enzyme of polyamine biosynthetic pathway that has been reported to exist in *E*.*histolytica*, [73, 74] [70]. Putrescine has been linked to OS resistance and one of the proposed mechanism of OS resistance is based on its polycationic nature that enables it to couple with nucleic acids and membrane phospholipids. Putrescine is also free radical scavenger and an antioxidant [75]. Putrescine probably plays the same antioxidant role in *E*. *histolytica* but in absence of an efficient inhibitor of *E*. *histolytica*'s ODC (α-difluoromethylornithine, which is a potent irreversible ODC inhibitor in many organisms) is not effective against *E*. *histolytica* ODC [70]), this hypothesis cannot be directly tested.

We found a very weak overlap between the results of this OX-RAC analysis of OXs and the results of an transcriptomics analysis of oxidatively stressed trophozoites [7]. This weak overlap indicates that the parasite's response to OS occurs at two different levels. One level is protein oxidation and the second level is a global change in gene expression which is characterized by the expression of general stress response-related proteins, such as heat shock proteins (HSPs) [8]. Another explanation is that oxidized proteins are not immediately expressed to replenish the parasite's cellular needs but are recycled through reduction processes. Such recycling may be done by EhPDI, an oxidoreductase that catalyzes oxidation, reduction and isomerization of disulfide bonds in polypeptide substrates [76]. A third explanation is that proteins which are encoded by OS response genes are resistant to inactivation by oxidation and for this reason they were not detected by OX-RAC. This explanation appears not to apply to chaperone-like heat-shock proteins or ubiquitin-conjugating enzymes because their expression is upregulated by OS [7] and oxidation inhibits their activity [77, 78].

We identified 21 common OX and SNO proteins (Table 3). With the exception of the Gal/GalNac lectin (this work), the effect of oxidation or S-nitrosylation on their activity/function has yet to be determined. This effect may be complex and often antagonist. For example, the activity of mammalian protein disulfide isomerase [79] and prokaryotic iron-containing superoxide dismutase [80] is inhibited when they are S-nitrosylated. In contrast, S-nitrosylation of superoxide dismutase and protein phosphatase 1B prevents their inactivation by OS [81, 82]. Thioredoxin must be S-nitrosylated for it to be an efficient antioxidant in plants [83]. It has been suggested that OXs and SNO proteins are mediators between stress pathways that are induced by OS and NS [84]. A good candidate for such mediator function is a member of the Ras-family GTPase, one of the common OX and SNO proteins which we identified in this study. Ras-family GTPases are involved in cell proliferation and their activity in mammalian is regulated by both S-nitrosylation [85] and oxidation [86].

To conclude, we inform on the presence of many novel OXs in oxidatively stressed *E*. *histolytica* trophozoites. Of these oxidized proteins, we discovered that (a) protein oxidation can regulate the activity of an important virulence factor of *E*.*histolytica*, namely Gal/GalNAc lectin, and (b) a protective role for arginase against OS. OX-RAC detects, enriches, and identifies OXsby detecting their oxidized cysteine residues, and it is possible that some of the OXs may not have been detected because of oxidation of other amino acid residues, such as methionine and tyrosine [87]. Finally, we envisage that these results will pave the way for further studies on the activity of OXs in *E*. *histolytica*.

## Supporting Information

S1 Table List of all OXs that were enriched by RAC in three independent experiments. (XLSX)

S2 Table List of OXs that were enriched at least two times or more by RAC in three independent experiments. (XLSX)

S3 Table Description of the parameters that are given in S1 and S2 Tables. (DOCX)

S1 Fig Viability of *E*. *histolytica* trophozoites which were exposed to different concentrations of H_2_O_2_ for 60 minutes at 37°C. Data are expressed as the mean ± standard deviation of three independent experiments that were repeated twice.
(JPG)
