# Supplementary material for: Proteomic Identification of Oxidized Proteins in Entamoeba histolytica by Resin-Assisted Capture: Insights into the Role of Arginase in Resistance to Oxidative Stress
Source: PLoS Negl Trop Dis. 2016 Jan 6;10(1):e0004340. doi: 10.1371/journal.pntd.0004340 (PMC4703340; doi:10.1371/journal.pntd.0004340)
Supplement: S3 Table — (DOCX) [file pntd.0004340.s003.docx]

Proteins Page Parameters

[This table](mk:@MSITStore:C:\\PROGRA~1\\Thermo\\DISCOV~1.3\\System\\Release\\THERMO~1.CHM::/Proteins%20Page%20Parameters.htm#1460389) describes the parameters available on the Proteins page.

| Proteins page parameters | |
| --- | --- |
| Feature | Description |
| Accession | Displays by default the unique identifier assigned to the protein by the FASTA database used to generate the report. |
| Description | Provides the name of the protein exclusive of the identifier that appears in the Accession column. This description appears in the table by default. |
| Coverage | Displays by default the percentage of the protein sequence covered by identified peptides. |
| # Proteins | Displays the number of identified proteins in the protein group of a master protein. This number is the same as that displayed in the Protein Group Members view when you choose Search Report > Show Protein Group Members View (see [this figure](mk:@MSITStore:C:\PROGRA~1\Thermo\DISCOV~1.3\System\Release\THERMO~1.CHM::/Interpreting%20Your%20Results%20with%20the%20Protein%20Group%20Members%20View.htm#1356604)). |
| # Unique Peptides | Displays the number of peptide sequences unique to a protein group. |
| # Peptides | Displays the number of distinct peptide sequences in the protein group. |
| # PSMs | Displays the total number of identified peptide sequences (peptide spectrum matches) for the protein, including those redundantly identified. |
| # AAs | Shows by default the sequence length of the protein. |
| MW [kDa] | Displays the calculated molecular weight of the protein. The Proteome Discoverer application calculates the molecular weight without considering PTMs.  Separating proteins by molecular weight can be one of the steps in two-dimensional gel electrophoresis. You can use the protein’s molecular weight as a rough constraint to estimate whether it is reasonable to identify a particular protein in a certain fraction that was searched. |
| calc. pI | Displays the theoretically calculated isoelectric point, which is the pH at which a particular molecule carries no net electrical charge.  The amino acids that make up proteins can be positive, negative, neutral, or polar in nature, and together they give a protein its overall charge. At a pH below their isoelectric point, proteins carry a net positive charge; at a pH above their isoelectric point, they carry a net negative charge. Gel electrophoresis can then separate proteins according to their isoelectric point (overall charge) with a polyacrylamide gel, using a technique called isoelectric focusing, which uses a pH gradient to separate proteins. Isoelectric focusing is also the first step in two-dimensional gel polyacrylamide gel electrophoresis.  When you have searched the fractions resulting from isoelectric focusing, you can use the calc. pI value to estimate whether you might expect to find a particular protein in the given fraction. |
| Area | Displays the average area of the three unique peptides with the largest peak area. |

Clicking the plus (+) sign next to any protein opens the column parameters for the associated peptides. For descriptions of these parameters, see [Peptides Page Parameters](mk:@MSITStore:C:\PROGRA~1\Thermo\DISCOV~1.3\System\Release\THERMO~1.CHM::/Peptides%20Page%20Parameters.htm#1083590).
