# Supplementary figures and images for: Proteomic Identification of Oxidized Proteins in Entamoeba histolytica by Resin-Assisted Capture: Insights into the Role of Arginase in Resistance to Oxidative Stress
Source: PLoS Negl Trop Dis. 2016 Jan 6;10(1):e0004340. doi: 10.1371/journal.pntd.0004340 (PMC4703340; doi:10.1371/journal.pntd.0004340)

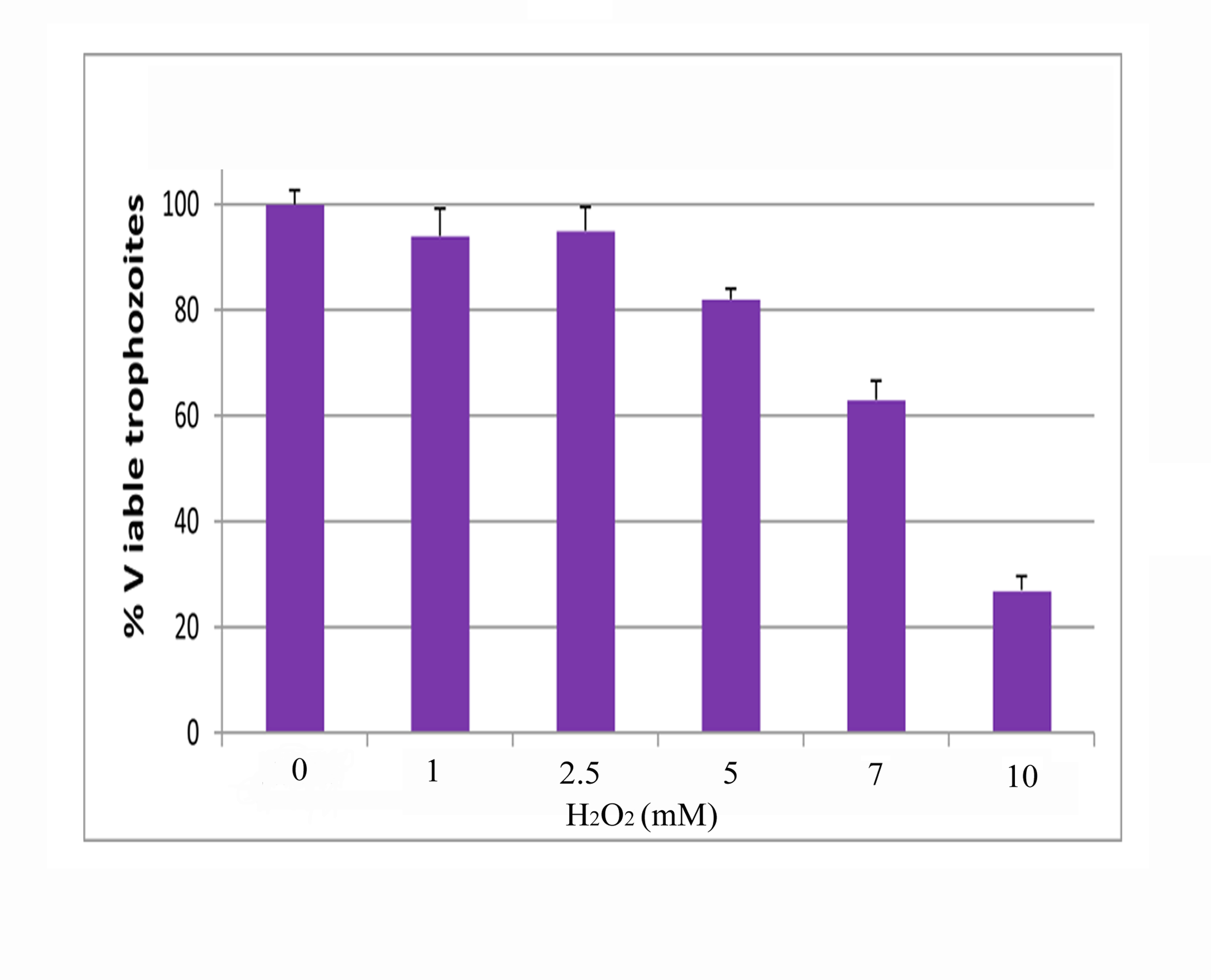

Supplement: S1 Fig — Data are expressed as the mean ± standard deviation of three independent experiments that were repeated twice. (JPG) [file pntd.0004340.s004.jpg]
